# Middle-term prognosis in patients with ulcerative colitis who achieved clinical and endoscopic remission by budesonide rectal foam

**DOI:** 10.1371/journal.pone.0220413

**Published:** 2019-08-05

**Authors:** Makoto Naganuma, Fumihito Hirai, Kiyonori Kobayashi, Kenji Watanabe, Ken Takeuchi, Nobuo Aoyama, Hiroshi Nozawa, Satoshi Motoya, Toshihide Ohmori, Akio Harada, Yushi Nagai, Takayuki Abe, Yoji Yamada, Katsutoshi Inagaki, Naoki Shimizu, Takanori Kanai, Mamoru Watanabe, for the ESCORT study Group

**Affiliations:** 1 Division of Gastroenterology and Hepatology, Department of Internal Medicine, Keio University School of Medicine, Tokyo, Japan; 2 Department of Gastroenterology and Medicine, Fukuoka University Faculty of Medicine, Fukuoka, Japan; 3 Department of Research and Development Center for New Medical Frontiers, Kitasato University School of Medicine, Kanagawa, Japan; 4 Department of Intestinal Inflammation Research, Hyogo College of Medicine, Nishinomiya, Japan; 5 Division of Gastroenterology and Hepatology, IBD Center, Tsujinaka Hospital Kashiwanoha, Chiba, Japan; 6 Gastrointestinal Endoscopy and Inflammatory Bowel Disease Center, Aoyama Medical Clinic, Hyogo, Japan; 7 Matsushima Clinic, Kanagawa, Japan; 8 IBD Center, Sapporo Kosei General Hospital, Hokkaido, Japan; 9 Ohmori Toshihide Gastro-Intestinal Clinic, Ageo, Japan; 10 Yokoyama Memorial Hospital, Nagoya, Japan; 11 Data Management Office, Clinical and Translational Research Center, Keio University School of Medicine, Tokyo, Japan; 12 School of Data Science, Yokohama City University, Yokohama, Japan; 13 Keio University School of Medicine, Tokyo, Japan; 14 Clinical Development Department, EA Pharma Co., Ltd., Tokyo, Japan; 15 Medical Science Group, Medical Department, EA Pharma Co., Ltd., Tokyo, Japan; 16 Medical Research Department, Kissei Pharmaceutical Co., Ltd., Tokyo, Japan; 17 TMDU Advanced Research Institute, Tokyo Medical and Dental University, Tokyo, Japan; Kurume University School of Medicine, JAPAN

## Abstract

**Background:**

Budesonide foam is effective in inducing clinical remission in ulcerative colitis (UC) patients with active proctosigmoiditis. The aim of this study was to evaluate the duration of remission and predictors of relapse in UC patients who achieved clinical remission and mucosal healing by 6-week treatment with topical budesonide.

**Methods:**

This is a retrospective, observational, multicenter study with a 2-year follow-up period. UC patients who were treated with budesonide foam in phase 2 or phase 3 clinical trials and achieved both clinical remission and mucosal healing were enrolled.

**Results:**

Among 84 patients who met the eligibility criteria, 60 participated in the study. Eighteen of the 60 patients (30.0%; 95% confidence interval [CI]: 18.9–43.2) experienced no relapse (i.e., maintenance of remission) during the 2-year follow-up period. The median relapse-free survival time was 0.82 years (95% CI: 0.51–1.52). Of 37 patients with a Mayo endoscopic subscore of 0 after inducing remission with budesonide foam, 25 (67.6%) relapsed within 2 years. Patients with a disease duration of <1 year experienced a worse clinical outcome than patients with a disease duration of >5 years, and the hazard ratio was 2.38 (95% CI: 1.04–5.45).

**Conclusion:**

This is the first study to evaluate the short- to middle-term prognosis in UC patients who achieved mucosal healing with topical preparations. After inducing remission by budesonide foam, treatment for maintaining remissions and strict follow-up may be needed for patients with shorter disease duration.

## Introduction

Ulcerative colitis (UC) is a nonspecific chronic inflammatory bowel disease, which is a progressive disease that follows a relapsing and remitting course [1]. Treatment for patients with UC is to reduce symptoms and introduce remission as soon as possible during the active stage. After the introduction of remission, management is required for its maintenance. Oral and topical 5-aminosalicylic acid (5-ASA) is a standard treatment for the induction and maintenance of remission in mild to moderate UC [2–4]. Corticosteroids are also a mainstay of induction therapy for UC patients in the active stage in the real world [5]. However, dose reduction and discontinuation of corticosteroids are recommended after inducing remission.

Recently, routine endoscopic assessment of disease activity and mucosal healing has emerged as a key goal of UC therapy as part of a treat-to-target strategy in clinical practice [6, 7]. Frøslie et al. reported that mucosal healing was significantly associated with a low risk of future colectomy, less inflammation after 5 years, and decreased future steroid treatment [8]. Furthermore, it has been found that patients who achieve endoscopic remission have improved outcomes, including reduced hospitalizations [9], use of immunomodulators [9], long-term maintenance therapy with systemic corticosteroids [10–14], and risk of colon cancer [15].

Budesonide is a synthetic corticosteroid with a highly potent anti-inflammatory effect at the topical administration site [16] and fewer systemic glucocorticoid-related side effects than other corticosteroids [17–19]. Budesonide foam is a rectally administered second-generation corticosteroid. In Japan, budesonide rectum foam (RECTABUL) was approved in 2017 for the treatment of mild to moderate UC based on phase 2 and phase 3 clinical trials in Japan. Mild to moderate UC patients in which the site of active inflammation is localized from the rectum to the sigmoid colon were enrolled in randomized, double-blind, placebo-controlled clinical trials, and efficacy of budesonide 2 mg foam was evaluated by endoscopic complete mucosal healing of the distal lesion at week 6. In phase 2 clinical trial, twice daily administration (BID) of budesonide foam for 6 weeks led to 46.4% of complete mucosal healing (Mayo endoscopic subscore [MES] = 0) and was superior to both once daily administration (QD) (23.6%) and placebo (5.6%). The proportion of MES ≤1 in the BID group and QD groups were 76.8% and 69.1%, respectively [20]. The phase 3 clinical trial demonstrated that 32.8% of complete mucosal healing and 40.6% of clinical remission were achieved by budesonide foam BID for 6 weeks in mild to moderate UC patients, which were significantly higher than those by placebo without any safety concerns [21].

To date, there has been no observational study reporting the duration of remission in patients who achieved mucosal healing by topical preparations. Therefore, we planned this observational study to evaluate the duration of remission, and predictors of relapse in UC patients who achieved clinical remission and mucosal healing (MES ≤ 1) by treatment with budesonide foam.

## Materials and methods

### Study design and patients

This examination of remission period in UC patients led to mucosal remission by induction therapy with budesonide rectal foam (the ESCORT study) is a retrospective, observational, multicenter, and a non-interventional study conducted at 23 sites in Japan between March 2018 and December 2018 (trial registration: UMIN 000031983). The protocol was approved by an independent ethics committee or the institutional review board of each institution.

**Fig 1:**
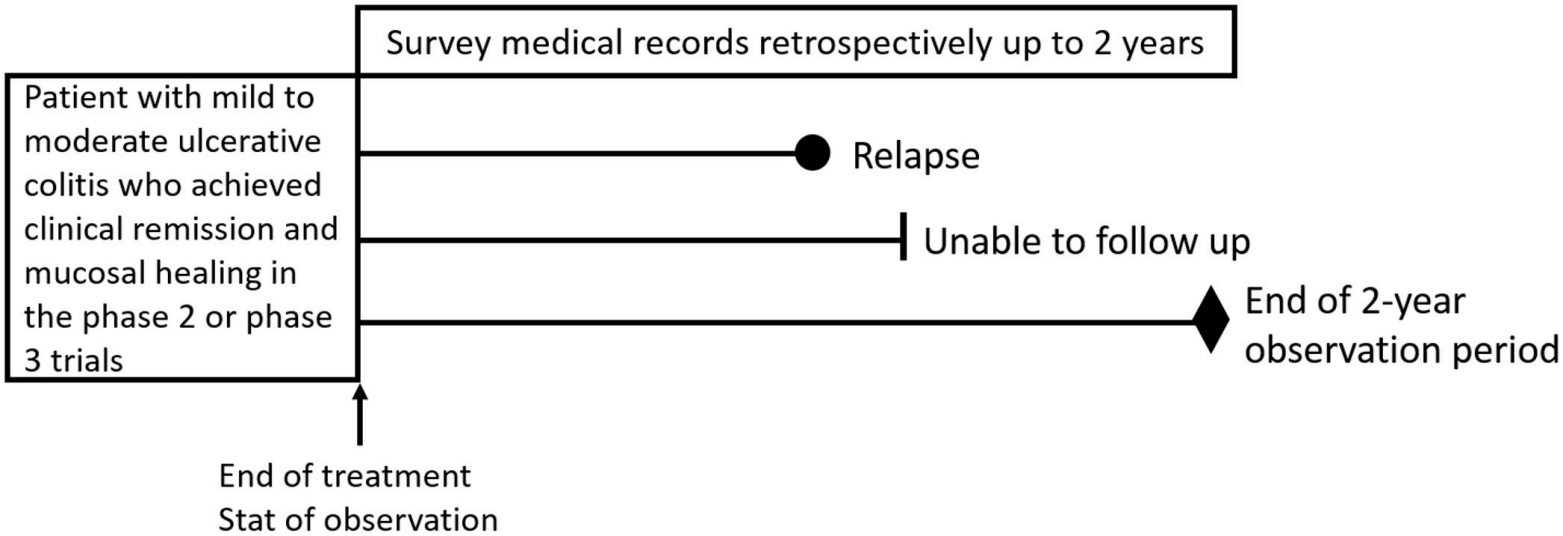
Study design.

UC patients who were treated with budesonide foam in the phase 2 or phase 3 clinical trials in Japan, and achieved clinical remission and mucosal healing were enrolled in this ESCORT study. Inclusion criteria were a Mayo bleeding subscore of 0; a Mayo stool frequency subscore of 0 or decrease in stool frequency subscale score ≥ 1 from week 0; and an MES ≤ 1 assessed at the central colonoscopy evaluation committee. Patients who had no endoscopic subscore at the end of treatment were excluded from the study, even if they were treated with budesonide foam in previous clinical trials. The observation period lasted until relapse of the disease or lost to follow-up during two years after the end of treatment with budesonide foam in the phase 2 or phase 3 clinical trials (Fig 1). The observation was terminated at the point when two years elapsed from the end of treatment with budesonide foam or unable to follow up with a patient. Relapse was defined as the onset of rectal bleeding, excluding hematochezia due to infectious enteritis, or the start of treatment with induction therapy for UC. The treatment period with budesonide foam was only 6 weeks in our phase 2 and phase 3 trials, and no patients received any budesonide foam during the follow-up period. However, most patients were treated with concomitant medication for maintaining remission. Table 1 describes concomitant medication at the baseline of the study.

**Table 1:** Patient demographics and baseline characteristics (analysis set).

Sex, n (%)		
Male		35 (58.3)
Female		25 (41.7)
Age (years), Mean (SD)		41.4 (12.0)
Body weight, (kg), Mean (SD)		59.9 (13.7)
Mayo endoscopic subscore at end of treatment with budesonide foam, n (%)		
Score 0		37 (61.7)
Score 1		23 (38.3)
Regimen of budesonide foam, n (%)		
Once daily		18 (30.0)
Twice daily		42 (70.0)
Smoking habit, n (%)		
Yes		1 (1.7)
No		59 (98.3)
Duration of disease (years), n (%)		
<1		11 (18.3)
≥1 to <5		22 (36.7)
≥5		27 (45.0)
Clinical course at the phase 2 or phase 3 trials, n (%)		
First attack		5 (8.3)
Relapsing-remitting		55 (91.7)
Extension of Disease, n (%)		
Proctitis		27 (45.0)
Left-sided colitis		28 (46.7)
Pancolitis		5 (8.3)
Extension of Disease 2, n (%)		
Proctitis		27 (45.0)
Left-sided colitis or pancolitis		33 (55.0)
Extension of Disease 3, n (%)		
Proctitis or left-sided colitis		55 (91.7)
Pancolitis		5 (8.3)
Severity, n (%)		
Mild		25 (41.7)
Moderate		35 (58.3)
Concomitant medication at start of the observation study, n (%)		
Oral 5-ASA	No	11 (18.3)
	< Max dose	22 (36.7)
	Max dose ^a^	27 (45.0)
Topical 5-ASA	No	43 (71.7)
	Yes	17 (28.3)
Topical corticoid^b^	No	54 (90.0)
	Yes	6 (10.0)

5-ASA, 5-Aminosalicylic acid; max, maximum; SD, standard deviation

^a^ Max dose of 5-ASA: Pentasa tablet and Salazopyrin tablet (4000 mg), Asacol tablet (3600 mg)

^b^ Suppository or enema. One case used corticoid injection.

### Data assessment

All data were collected from June 2018 to November 2018 in each institution. Demographic characteristics at the start of the clinical trials included sex, age, weight, smoking status, disease duration, clinical course (first attack or relapse/remitting), concomitant medications, disease type, and severity of the disease (defined by modified Truelove and Witt’s definition [22] Clinical symptoms including rectal bleeding, mucus in stool, abdominal pain, and diarrhea occurred during the observation period were recorded with the date of the examination. Diarrhea was recorded with daily frequency. Maintenance therapy including medications was also recorded. All data were collected through electronic case report forms. The primary endpoint was the duration of remission from the end of treatment with budesonide foam. Secondary endpoints were to find predictors associated with duration of remission in patient characteristics including an MES (score = 0 and score = 1) at the start of this study regimen of budesonide foam (once daily and twice daily), and type of disease (proctitis, left-sided colitis, and pancolitis).

### Statistical analysis

All data from each institution were sent to the central data center for data management (Clinical and Translational Research Center, Keio University School of Medicine) before statistical analysis. Demographic factors and baseline characteristics of study participants were summarized. The primary full analysis set (FAS) included all study participants who had at least one follow-up data. The primary endpoint was duration of remission. The relapse-free survival was estimated with the Kaplan–Meier method, and a nonparametric estimate for median relapse-free survival time and its 95% confidence interval (CI) were calculated. As exploratory analyses in terms of prognostic factors for the relapse, the hazard ratio (HR) was estimated with the use of the Cox proportional hazards model. The log-rank test was applied to compare the relapse-free survival between the groups. The characteristics of patients, an MES (score = 0 or 1) at the start of the observation, the dosing regimen of budesonide foam (QD or BID), and the type of disease (proctitis, left-sided colitis, and pancolitis) were summarized in the relapse-free and the relapse groups. The distribution of each variable was compared between the two groups using a two-sample *t*-test or Fisher’s exact test. The 95% CIs for proportion were calculated using the Clopper–Pearson method. The significance level for each test was 5% (two-tailed). All analyses were performed using SAS version 9.4 (SAS Institute, Cary, NC, USA).

### Ethical considerations

The study was conducted in accordance with the Declaration of Helsinki and the Ethical Guidelines for Medical and Health Research Involving Human Subjects [23]. Informed consent was obtained in accordance with the Ethical Guidelines for Medical and Health Research Involving Human Subjects.

## Results

### Patient disposition and baseline characteristics

**Fig 2:**
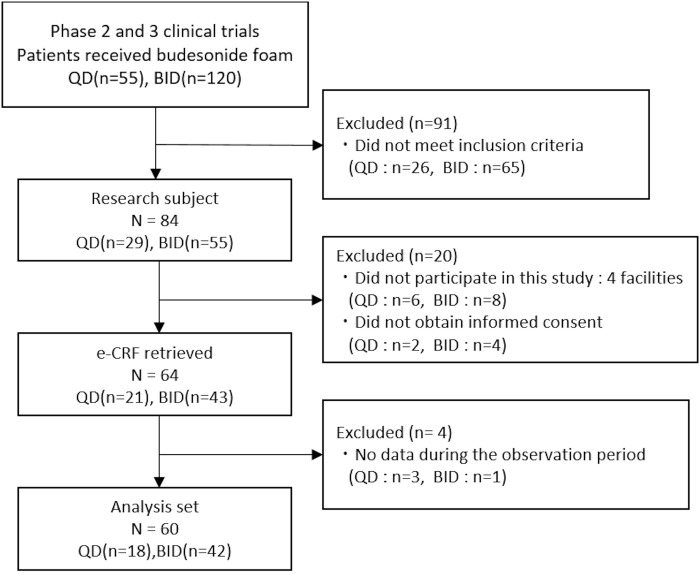
Patient disposition. eCRF, electronic case report form; BID, twice daily administration; QD, once daily administration.

The numbers of UC patients who received budesonide foam in the clinical trials in Japan were 55 in the QD and 56 in the BID regimens in the phase 2 study and 64 in the BID regimen in the phase 3 study. Among 84 patients who met the eligibility criteria, 14 patients who met the eligibility criteria were excluded because of no participation in this study (four institutions) and 6 patients who did not give informed consent were also excluded. Finally, 64 patients (40 patients in phase 2 and 24 in phase 3 studies) were enrolled in the ESCORT study. The FAS population consisted of 60 patients after excluding 4 patients with no follow-up data (Fig 2). Patient demographics and baseline characteristics are shown in Table 1. There were slightly more male patients (58.3%, *n* = 35) than female patients (41.7%, *n* = 25). At the end of treatment with budesonide foam in the former phase 2 or phase 3 clinical trials, 61.7% (*n* = 37) of patients had an MES of 0, and 38.3% (*n* = 23) had an MES of 1. Patients receiving QD and BID budesonide foam during the former clinical trials were 18 (30.0%) and 42 (70.0%), respectively. The numbers of patients with a disease duration of <1 year, ≥1 to <5 years, and ≥5 years were 11 (18.3%), 22 (36.7%), and 27 (45.0%), respectively. The disease types of UC were proctitis (*n* = 27, 45.0%), left-sided colitis (*n* = 28, 46.7%), and pancolitis (*n* = 5, 8.3%). The severity of disease of 25 (41.7%) patients was mild, and 35 (58.3%) was moderate.

### Primary endpoint

**Fig 3:**
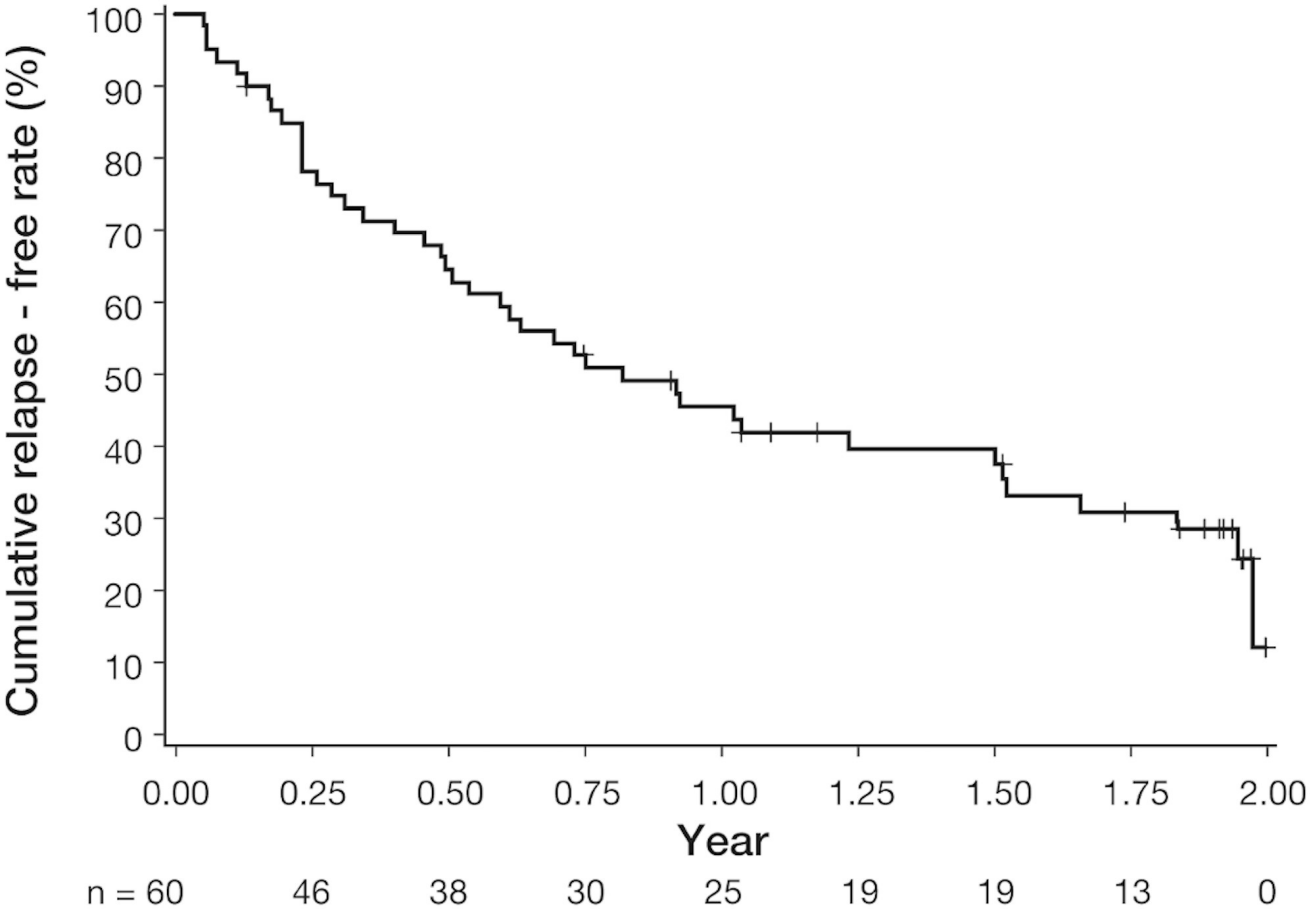
Kaplan–Meier plot for the relapse in patients with ulcerative colitis who achieved clinical remission and mucosal healing by treatment with budesonide foam for 6 weeks in the phase 2 or phase 3 clinical trials in Japan. The median survival time (year) without relapse was 0.82 (95% CI: 0.51 to 1.52).

Both clinical remission and mucosal healing were achieved in all 60 patients at baseline, and 18 (30.0%; 95% CI: 18.9% to 43.2%) did not experience relapse (maintenance of remission) during the 2-year observation period after the clinical trials. The Kaplan–Meier plot for the cumulative proportion of relapse-free survival is shown in Fig 3. The median relapse-free survival time was 0.82 years (95% CI: 0.51–1.52). The cumulative remission rates at 3, 6, and 12 months were 78.1%, 64.5%, and 45.5%, respectively.

### Secondary endpoints

**Fig 4:**
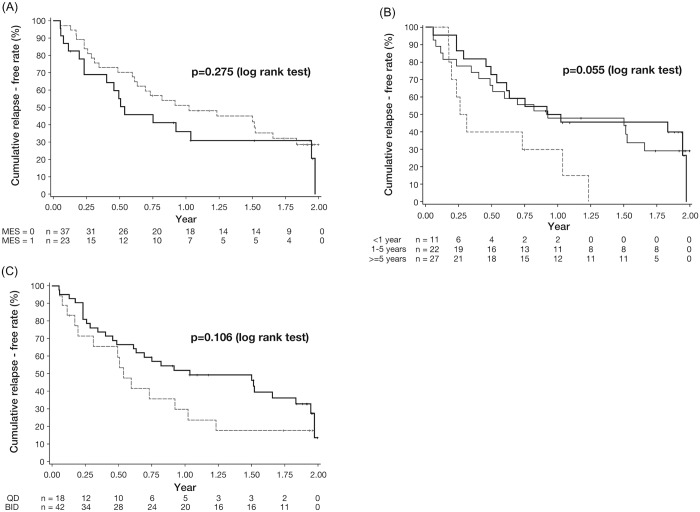
Kaplan–Meier plot for the relapse in patients with ulcerative colitis who achieved clinical remission and mucosal healing by treatment with budesonide foam in phase 2 or phase 3 clinical trials in Japan by a covariate. (A) endoscopic subscore: MES = 0 (broken line) and MES = 1 (solid line) at start of this study, each median survival time (year) was 1.02 (95% CI: 0.61 to 1.66) and 0.54 (95% CI: 0.23 to 1.95), respectively. There was no significant difference between the groups (log-rank test, P = 0.275). (B) The disease duration: duration of <1 year(broken line), duration of 1–5 years (solid line), duration of ≥5 years (thin solid line). Each median survival times (year) were 0.28 (95% CI: 0.17 to 1.03), 0.97 (95% CI: 0.49 to 1.97), 0.93 (95% CI: 0.40 to 1.66), respectively. There was no significant difference between Group 1, 2 and 3 (log-rank test, P = 0.055). (C) Patients receiving budesonide foam, QD (broken line) and BID (solid line), each median survival times (year) were 0.54 (95% CI: 0.19 to 1.02) and 1.03 (95% CI: 0.61 to 1.83), respectively. There was no significant difference between Group 1 and Group 2 (log-rank test, P = 0.106).

Table 2 provides the proportion of patients with or without relapse during the observation period by each covariate. None of the patients’ demographic, clinical, biological, or histological features, except body weight, was found to be a risk factor for relapse as mean body weight in the remission group was significantly higher than in the relapsing group (*P* = 0.014). Even patients with an MES of 0 after inducing remission with budesonide foam, 25 of 37 patients (67.6%) had relapses during 2 years, and the rates of remission were comparable between patients with an MES of 0 and an MES of 1 (Table 2; *P* = 0.773). The Kaplan–Meier plots are shown in Fig 4. The median survival years without relapse in patients with an MES of 0 and an MES of 1 were 1.02 (95% CI: 0.61 to 1.66) and 0.54 (95% CI: 0.23 to 1.95), respectively (log-rank test *P* = 0.275). The period from baseline to relapse was comparable between patients with an MES of 0 and patients with an MES of 1 (Fig 4A, Table 3). The median survival year without relapse in patients with disease duration<1 year (0.28, 95% CI: 0.17 to 1.03) tended to be shorter than that with >5 years (0.93, 95% CI: 0.40 to 1.66) (Fig 4B). However, there was no significant difference between the both groups (log-rank test *P* = 0.055). The median survival without relapse (years) in QD and BID was 0.54 (95% CI: 0.19 to 1.02) and 1.03 (95% CI: 0.61 to 1.83), respectively (Fig 4C). There was no significant difference between groups in any other covariates such as the dosing regimen of budesonide foam at the clinical trials (QD or BID; Table 3). Using the univariate Cox regression model, estimated HRs for body weight and disease duration <1 year were 0.97 (95% CI: 0.94–0.99, *P* = 0.017) and 2.38 (95% CI: 1.04–5.45, *P* = 0.040), respectively (Table 3).

**Table 2:** Proportion of patients with/without relapse during the observation period by covariates.

Covariates		Remission maintenance	Relapse	*P*-value ^a^
Sex, n (%)				
Male		13 (37.1)	22 (62.9)	0.253
Female		5 (20.0)	20 (80.0)	
Age (years), Mean (SD)		42.7 (11.4)	40.8 (12.3)	0.586
Body weight, (kg), Mean (SD)		66.4 (14.2)	57.2 (12.6)	0.014
Endoscopic subscore at end of treatment with budesonide foam, n (%)				
Score 0		12 (32.4)	25 (67.6)	0.773
Score 1		6 (26.1)	17 (73.9)	
Regimen of budesonide foam, n (%)				
Once daily		4 (22.2)	14 (77.8)	0.542
Twice daily		14 (33.3)	28 (66.7)	
Smoking habit, n (%)				
Yes		1 (100.0)	0 (0.0)	0.300
No		17 (28.8)	42 (71.2)	
Duration of disease (years), n (%)				
<1		2 (18.2)	9 (81.8)	0.706
≥1 to <5		7 (31.8)	15 (68.2)	
≥5		9 (33.3)	18 (66.7)	
Clinical course at the phase 2 or phase 3 trials, n (%)				
First attack		2 (40.0)	3 (60.0)	0.631
Relapsing-remitting		16 (29.1)	39 (70.9)	
Extension of Disease, n (%)				
Proctitis		10 (37.0)	17 (63.0)	0.075
Left-sided colitis		5 (17.9)	23 (82.1)	
Pancolitis		3 (60.0)	2 (40.0)	
Extension of Disease 2, n (%)				
Proctitis		10 (37.0)	17 (63.0)	0.397
Left-sided colitis or Pancolitis		8 (24.2)	25 (75.8)	
Extension of Disease 3, n (%)				
Proctitis or left-sided colitis		15 (27.3)	40 (72.7)	0.154
Pancolitis		3 (60.0)	2 (40.0)	
Severity, n (%)				
Mild		10 (40.0)	15 (60.0)	0.168
Moderate		8 (22.9)	27 (77.1)	
Concomitant medication at start of the observation study, n (%)				
Oral 5-ASA	No	4 (36.4)	7 (63.6)	0.870
	<Max dose ^b^	6 (27.3)	16 (72.7)	
	Max dose ^a^	8 (29.6)	19 (70.4)	
Topical 5-ASA	No	14 (32.6)	29 (67.4)	0.550
	Yes	4 (23.5)	13 (76.5)	
Topical corticoid ^c^	No	18 (33.3)	36 (66.7)	0.165
	Yes	0 (0.0)	6 (100.0)	

5-ASA, 5-Aminosalicylic acid; SD, standard deviation

^a^ Two-tailed *P*-value with Fisher’s exact test or two sample *t* test

^b^ Max dose of 5-ASA: Pentasa tablet and Salazopyrin tablet (4000 mg), Asacol tablet (3600 mg)

^c^ Suppository or enema. One case used corticoid injection.

**Table 3:** Estimated hazard ratio (HR) with Cox regression model.

Valuables		*P*-value ^a^	*P*-value ^b^	HR	95% CI
Sex	Female / male	0.095	0.100	1.68	(0.91, 3.11)
Age (df = 1)			0.681	0.99	(0.97, 1.02)
Body weight (df = 1)			0.017	0.97	(0.94, 0.99)
Mayo endoscopic subscore ^c^ at end of treatment with budesonide foam	Score = 1 / Score = 0	0.275	0.279	1.41	(0.76, 2.62)
Regimen of budesonide foam	Twice daily / once daily	0.106	0.111	0.59	(0.31, 1.13)
Smoking habit	No / Yes	NA	NA	NA	NA
Duration of disease (year)	<1 / ≥5	0.055	0.040	2.38	(1.04, 5.45)
	≥1 to <5 / ≥5		0.810	0.92	(0.46, 1.83)
Clinical course at the phase 2 or phase 3 trials	Relapsing-remitting / first attack	0.526	0.530	0.68	(0.21, 2.24)
Extension of Disease	Proctitis / pancolitis	0.350	0.358	1.99	(0.46, 8.66)
	Left-sided colitis / pancolitis		0.198	2.59	(0.61, 11.1)
Extension of Disease 2	Pancolitis + left-sided colitis / Proctitis	0.658	0.659	1.15	(0.62, 2.14)
Extension of Disease 3	Pancolitis / proctitis + left-sided colitis	0.240	0.255	0.44	(0.10, 1.82)
Severity	Modulate / mild	0.411	0.414	1.30	(0.69, 2.45)
Oral 5-ASA	No / max dose ^d^	0.869	0.704	1.18	(0.49, 2.84)
	<the max dose / max dose		0.833	0.93	(0.48, 1.81)
Topical 5-ASA	Yes /No	0.276	0.280	1.44	(0.74, 2.81)
Topical corticosteroid ^e^	Yes /No	0.143	0.151	1.90	(0.79, 4.57)

5-ASA, 5-Aminosalicylic acid; CI, confidence interval; df, degrees of freedom; NA, not available

^a^ Two-tailed *P*-value with log rank test

^b^ Two-tailed *P*-value with univariate Cox regression

^c^ Endoscopic subscore in a Modified Mayo Disease Activity Index subscore.

^d^ Max dose of 5-ASA: Pentasa tablet and Salazopyrin tablet (4000 mg), Asacol tablet (3600 mg)

^e^ Suppository or enema. One case used corticoid injection.

The risk factor to predict relapse only in patients with an MES of 0 (n = 37) was also analyzed. Body weight (HR 0.96 (95% CI:0.93–0.99) and disease duration<1 year (vs. >5 years, HR 4.07 (95% CI:1.45–11.5) was found to be a risk factor for relapse n patients with an MES of 0 at baseline.

## Discussion

In this ESCORT study, short- to middle-term prognosis was analyzed in patients who achieved both clinical remission and mucosal healing by a 6-week treatment with budesonide foam. The risk factors for relapse during 2 years were also investigated in this study. We demonstrated that 30% of patients maintained remission for 2 years when clinical remission was achieved by treatments of budesonide foam. Interestingly, even complete endoscopic remission was achieved with budesonide foam, approximately 70% of patients with an MES of 0 had relapsed during the 2-year follow-up period. The median survival time without relapse in patients with an MES of 0 and an MES of 1 were 1.02 and 0.54 years, respectively. The median survival time without relapse in patients with QD and BID budesonide foam were 0.54 and 1.03 years, respectively. This investigation is the first study to clarify the prognosis in patients who achieved mucosal healing by topical preparations.

In the previous study [24], budesonide enema was effective in inducing clinical remission in UC patients with active proctosigmoiditis. However, treatment with the enema was not comfortable for some patients because of its poor tolerability and the high-volume application [24]. More recently, the efficacy of budesonide foam in patients with active UC has been confirmed in randomized control trials [20, 21, 25, 26]. In our recent phase 2 and phase 3 study [20, 21], we confirmed that the proportion of patients with MES of 0 in the budesonide foam group was significantly higher than those in the placebo group. In the present study, we aimed to investigate whether patients who achieved clinical remission and mucosal healing at week 6 by budesonide foam could maintain remission for longer-term periods. Particularly, we speculated that patients who achieved an MES of 0 at week 6 had fewer clinical relapses than patients who achieved an MES of 1 because previous studies indicated that complete mucosal healing had better outcomes than an MES of 1. Contrary to our expectations, most patients with an MES of 0 relapsed within 2 years after the clinical trials. The rate of relapse in patients with an MES of 0 was not different from that in patients with an MES of 1. The previous study indicated that prognosis was better if the duration of clinical remission treatment was longer [3, 11] while the duration of clinical remission was maximal at 6 weeks in our study. Endoscopic assessment at week 6 after treatment with budesonide foam may not be adequate for predicting prognosis.

The treatment period with budesonide foam was only 6 weeks in our phase 2 and phase 3 trials, and no patients received any budesonide foam during the follow-up period. On the other hand, a previous study on maintenance of remission using oral 5-ASA indicated that patients with an MES of 0 had better outcomes than patients with an MES of 1 did [27]. In this study, patients continued to use 2.4 g of 5-ASA daily during the followed-up period as maintenance therapy [28]. Therefore, the continuous use of the same medication after induction treatment may be useful for better prognosis. Both the safety and efficacy of long-term administration of budesonide for patients with asthma has been confirmed [29], and long-term administration of budesonide is being investigated. Although the safety and efficacy of long-term use of budesonide foam have not been confirmed, it may be better to use budesonide foam beyond 6 weeks for patients with risk factors for relapse even after an MES of 0 was achieved.

Our study also confirmed that lower body weight and a shorter disease duration was a risk factor for clinical relapse. Inadequate treatment may lead to clinical relapse in case of the shorter disease duration. Because patients with disease duration <12 months was a risk factor for relapse after inducing remission with budesonide foam in the present study, the strict follow-up and confirmation of adherence to treatment for maintaining remission should be required for these patients. It is not clear why lower body weight at baseline was a predictor for clinical relapse in this study. Insufficient nutrition even in patients with clinical remission may be associated with weight loss, which may lead to relapse.

Our study has some limitations. First, it is a retrospectively conducted study and has a relatively small sample size. Second, the definition of relapse was only clinically assessed; objective biomarkers, such as fecal calprotectin were not used in our study. However, few studies have investigated the prognosis of patients who achieved both clinical remission and mucosal healing with topical therapy. The results of our study included valuable information for medical treatment after induction of remission with budesonide foams.

In conclusion, the relapse rate was relatively high during the 2-year follow-up period, even in patients who achieved complete endoscopic healing by 6-week treatment with budesonide foam in the previous clinical trials. The risk factors for relapse were shorter disease duration in patients whose remission was induced with budesonide foam. Treatment for maintaining remission, such as continuous use of topical therapy and strict follow-up may be needed for patients with risk factors for relapse.

## Supporting information

S1 Table Estimated hazard ratio (HR) with Cox regression model inpatients with Mayo endoscopic score of 0 at baseline. (PDF)

S1 File STROBE checklist. (DOC)

S2 File ESCORT data. (XLSX)
