# Expression of Concern: Middle-term prognosis in patients with ulcerative colitis who achieved clinical and endoscopic remission by budesonide rectal foam

**DOI:** 10.1371/journal.pone.0280122

**Published:** 2023-01-11

**Authors:** The PLOS ONE Editors

The Competing Interests statement for this article [1] states that one of the authors received grant funding from the Smoking Research Foundation, which according to [2] has received financial support from the tobacco industry. It is unclear whether Smoking Research Foundation grant funding contributed to the author’s salary or research costs during the period when the study reported in [1] was conducted. The foundation is not listed as a primary funder for this study in the article’s Funding Statement.

In light of this issue the *PLOS ONE* article [1] may not comply with the journal’s policy on Funding from Tobacco Companies [3] which was implemented in 2010.

Therefore, the *PLOS ONE* Editors issue this Expression of Concern.

We regret that this concern was not identified and addressed prior to the article’s [1] publication.
