# Supplementary material for: Middle-term prognosis in patients with ulcerative colitis who achieved clinical and endoscopic remission by budesonide rectal foam
Source: PLoS One. 2019 Aug 5;14(8):e0220413. doi: 10.1371/journal.pone.0220413 (PMC6681969; doi:10.1371/journal.pone.0220413)
Supplement: S1 Table — (PDF) [file pone.0220413.s001.pdf]

Valuables		*P*-value [a]	*P*-value [b]	HR	95% CI
Sex	Female / male	0.154	0.159	1.76	(0.80, 3.89)
Age (df = 1)			0.375	0.98	(0.95, 1.02)
Body weight (df = 1)			0.019	0.96	(0.93, 0.99)
Regimen of budesonide foam	Twice daily / once daily	0.345	0.357	0.66	(0.28, 1.59)
Smoking habit	No / Yes	NA	NA	NA	NA
Duration of disease (year)	<1 / ≥5	0.016	0.008	4.07	(1.45, 11.5)
	≥1 to <5 / ≥5		0.505	1/37	(0.54, 3.49)
Clinical course at the phase 2 or phase 3 trials	Relapsing-remitting / first attack	0.747	0.748	0.79	(0.18, 3.40)
Extension of Disease	Proctitis / pancolitis	0.071	0.992	NA	NA
	Left-sided colitis / pancolitis		0.992	NA	NA
Extension of Disease 2	Pancolitis + left-sided colitis / Proctitis	0.893	0.893	1.06	(0.48, 3.40)
Extension of Disease 3	Pancolitis / proctitis + left-sided colitis	0.040	0.992	NA	NA
Severity	Modulate / mild	0.500	0.501	1.32	(0.59, 2.94)
Oral 5-ASA	No / max dose [d]	0.564	0.546	1.44	(0.44, 2.94)
	<the max dose / max dose		0.550	0.77	(0.33, 1.82)
Topical 5-ASA	Yes /No	0.779	0.779	0.88	(0.35, 2.20)
Topical corticosteroid [e]	Yes /No	0.191	0.191	1.93	(0.71, 5.29)

5-ASA, 5-Aminosalicylic acid; CI, confidence interval; df, degrees of freedom; NA, not available

a. Two-tailed *P*-value with log rank test
b. Two-tailed *P*-value with univariate Cox regression
c. Endoscopic subscore in a Modified Mayo Disease Activity Index subscore.
d. Max dose of 5-ASA: Pentasa tablet and Salazopyrin tablet (4000 mg), Asacol tablet (3600 mg)
e. Suppository or enema. One case used corticoid injection.
